# Cigarette Smoke Induced Lung Barrier Dysfunction, EMT, and Tissue Remodeling: A Possible Link between COPD and Lung Cancer

**DOI:** 10.1155/2019/2025636

**Published:** 2019-06-24

**Authors:** Wei Hou, Siyi Hu, Chunyan Li, Hanbin Ma, Qi Wang, Guangping Meng, Tingting Guo, Jie Zhang

**Affiliations:** ^1^Department of Respiratory and Critical Care Medicine, The Second Hospital of Jilin University, Changchun 130041, China; ^2^CAS Key Laboratory of Bio-Medical Diagnostics, Suzhou Institute of Biomedical Engineering and Technology, Chinese Academy of Science, Suzhou 215163, China

## Abstract

Chronic obstructive pulmonary disease (COPD) and lung cancer, closely related to smoking, are major lung diseases affecting millions of individuals worldwide. The generated gas mixture of smoking is proved to contain about 4,500 components such as carbon monoxide, nicotine, oxidants, fine particulate matter, and aldehydes. These components were considered to be the principle factor driving the pathogenesis and progression of pulmonary disease. A large proportion of lung cancer patients showed a history of COPD, which demonstrated that there might be a close relationship between COPD and lung cancer. In the early stages of smoking, lung barrier provoked protective response and DNA repair are likely to suppress these changes to a certain extent. In the presence of long-term smoking exposure, these mechanisms seem to be malfunctioned and lead to disease progression. The infiltration of inflammatory cells to mucosa, submucosa, and glandular tissue caused by inhaled cigarette smoke is responsible for the destruction of matrix, blood supply shortage, and epithelial cell death. Conversely, cancer cells have the capacity to modulate the proliferation of epithelial cells and produce of new vascular networks. Comprehension understanding of mechanisms responsible for both pathologies is necessary for the prevention and treatment of COPD and lung cancer. In this review, we will summarize related articles and give a glance of possible mechanism between cigarette smoking induced COPD and lung cancer.

## 1. Introduction

Chronic obstructive pulmonary disease (COPD) is commonly associated with prolonged exposure to toxic aerosol [1, 2]. The pathology of COPD is generally featured by persistent airflow limitation caused by chronic inflammation of the airways [3]. To date, it has been well acknowledged that COPD consists of two parts: the partial obstruction of air way ventilation and obliteration of the terminal alveolus in the peripheral regions of lung [4]. Cigarette smoking (CS) is the principle factor driving the pathogenesis and progression of pulmonary diseases [5, 6]. The generated gas mixture of smoking is proved to contain about 4,500 components such as carbon monoxide, nicotine, oxidants, fine particulate matter and aldehydes. These components were considered to be closely related to the onset of COPD [7]. Meanwhile, smoking and aberrant epithelial responses are risk factors for COPD and lung cancer [8]. A large proportion of lung cancer patients showed a history of COPD, which demonstrated that there might be close relationship between COPD and lung cancer [9]. Besides, COPD patients showed a 6-fold increase in the risk of developing lung cancer compared to the smokers [10]. In an epidemiological survey, adenocarcinoma predominates in Global Initiative for Chronic Obstructive Lung Disease (GOLD) Stage I while squamous cell carcinoma is the most frequent in GOLD Stages II and III [11]. Nevertheless, COPD or emphysema was not remarkably associated with cancer mortality [9]. In pathological view, the main histological changes of COPD are epithelial remodeling and subepithelial fibrosis of small airways [12]. The infiltration of inflammatory cells to mucosa, submucosa, and glandular tissue caused by inhaled cigarette smoke is responsible for the destruction of matrix, blood supply shortage, and epithelial cell death. Conversely, cancer cells have the capacity to modulate the proliferation of epithelial cells and produce of new vascular networks [13]. Up to now, increasing interests have been given to the possible link between cellular apoptosis and uncontrolled proliferation. In this review, we will summarize related article and give a glance of possible link between CS-induced COPD and lung cancer in order to provide a theoretical basis for the subsequent studies on the prevention and treatment of CS-induced COPD associated lung cancer.

## 2. CS Increased Alveolar-Capillary Barrier Permeability and Inflammation

**Figure 1:**
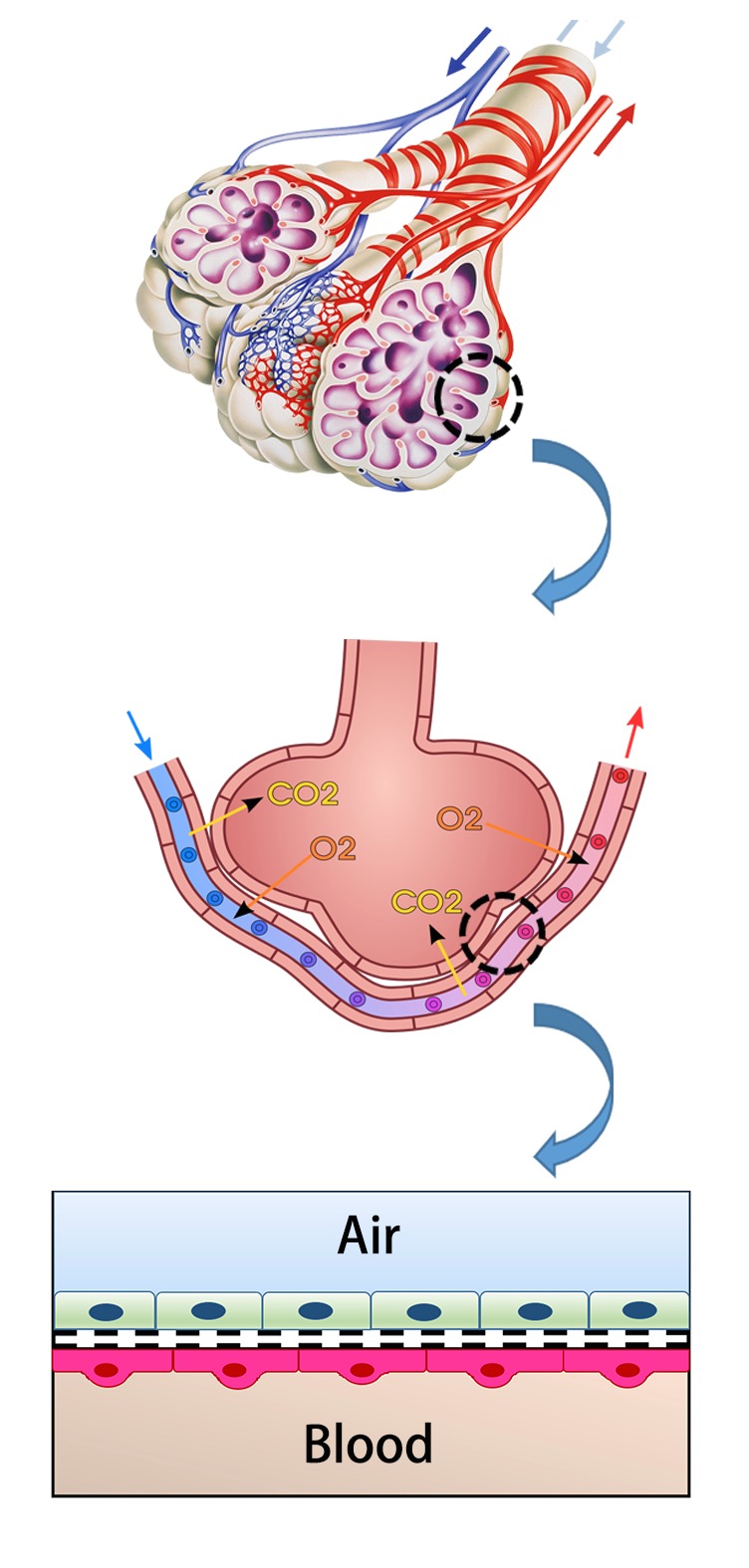
Simplified diagrammatic look of air-blood barrier.

The alveolar-capillary barrier, also known as lung air-blood barrier, acts as the place for O_2_ and CO_2_ exchange and the safeguard to bear the brunt of inhaled irritants. It is essentially composed of alveolar epithelium and capillary endothelium that is connected and/or separated by interstitial tissue of varying composition and width [14] (Figure 1). The airway epithelium was localized at the interface between the external environment and the lung parenchyma, which protected the subepithelial tissue from inhaled noxious stimuli and anaphylatoxin [15]. It has been widely accepted that lung endothelium is not a passive barrier to gas exchange. However, it is a critical mediator as it shows the ability of mediating inducible permeability in states of acute and chronic inflammation [16, 17]. Pulmonary edema is considered as an index of lung barrier dysfunction to a certain extent because alveolar epithelial-endothelial injury and inflammation are the basic elements for the pathological changes. The weight of lung of the smokers was higher than that of former smokers and none smokers, presumably to edema [18]. In the recent decades, the alveolar epithelial barrier was impaired in smokers [19]. To date, there are still disputes on whether CS exposure increases pulmonary endothelial permeability in humans. Loss of cellular junction and area of pulmonary endothelial denudation has been well documented in smokers with COPD under inspection with electron microscopy [20]; however, in a pilot study involving 5 healthy normal smokers and 5 nonsmokers, acute exposure to smoke may contribute to the decrease of pulmonary capillary barrier permeability to urea [21]. To our best knowledge, the toxic effects of CS are in a dose- and time-dependent manner. This indicated that there were diverse effects among acute, subacute, and chronic exposure. To further investigate the differences and underlying mechanisms, animal model and* in vitro* model were established and some meaningful results were obtained. Immense amounts of studies confirmed that both brief and subacute CS preexposure could increase susceptivity of lung injury in animal models. Moreover, these studies indicated that the increase of lung barrier permeability was resulted from both epithelial and endothelial cellular injuries [22–26].

In response to reduplicative smoke exposure, reduction in several apical junctional complex (AJC) has been observed in lung epithelium* in vitro* [27, 28]. Tight junctions (TJs) are the most apical paracellular structure which constitute a semipermeable barrier for solutes and ions [29]. Adhesion junctions (AJs), responsible for mediating cell-cell adhesion, were localized below the TJs [30]. The effects of CS on increased lung epithelium permeability were usually interpreted as direct damage to junction structure for both TJs and AJs [31]. TJs destruction is usually thought to be closely relevant to various kinds of tumorigenesis and metastasis such as colorectal cancer, pancreatic cancer, and prostatic cancer [32]. The integral transmembrane proteins are the fundamental adhesion molecule in charge of correct assembling of the TJ structure and controlling TJ functions. The regulation of cell adhesion was mediated by multiple signaling pathways, which were essential for cell growth, differentiation, gene expression, and motility [33]. In a recent study, Merikallio et al. reported that bronchial cell barrier was damaged by tobacco smoke though changing mRNA synthesis of claudins. Meanwhile, intense expression positivity of claudin 1 and claudin 4 was found more common in cancer samples of smokers and ex-smokers compared to nonsmokers [34]. In contrast, the downregulation in genetic level of* claudin 1*,* claudin 3*,* claudin 8*,* claudin 9*,* claudin 16*, and* occludens* was reported in smokers compared with nonsmokers. Additionally, the upregulation of* claudin 7 *and* claudin 10* was also reported [27]. Besides, an unexpected number of studies with* in vitro *models of CS exposure failed to detect the expression changes of TJ proteins such as the occludins and zonula occludens (ZOs) [35, 36]. This may relate to the fact that it is difficult to present the modeling chronic CS exposure and contributing factors (e.g., cytokines) induced by endothelium damage* in vivo* in short-term in vitro models. These results highlighted that a coculture model of epithelial cells and endothelial cells with the compatibility of prolonged CS exposure may lead to new discoveries in COPD and lung cancer.

Increase of lung barrier permeability might induce epithelial and endothelial cell injury. One of the most important obligations of endothelial cells is the regulation of vascular permeability. It is believed that endothelial cells from different tissues show different permeability [37], and lung endothelium is not a passive barrier to gas exchange. However, it is metabolically active and is a key mediator of inflammation [16]. CS exposure increases the surface expression of adhesion molecules, including ICAM-1, endothelial leukocyte adhesion molecule 1, E-selectin, and VCAM-1, as well as cytokines and chemokines, such as TNF-*α*, IL-6, and IL-1*β*, via NADPH oxidase-dependent NF-*κ*B transcriptional activation in endothelial cells [38]. It is also confirmed that CS exposure increases the permeability of pulmonary endothelial monolayer* in vitro* [39, 40]. The underlying mechanism is related to the regulation of AJs, microtubules [41], and focal adhesion complex [42]. These findings suggested that the injury of epithelium and endothelium may share some common pathways although there are some inherent differences in cellular connection and adhesion. Besides, targeting the vital factor could provide new solutions for the initial phase of CS-induced inflammation. Additionally, increased alveolar-capillary permeability and solubility of toxic molecule in blood will cause subsequent damage out of lungs. It could also mean that lung barrier dysfunction is the preliminary lesion for CS associated lesion in other organs. The transformation from chronic inflammation to cancer has long been extensively investigated in other organs or systems. Chronic exposure to toxic environmental agents is a risk factor in certain kinds of cancer, such as asbestos exposure for pleural mesothelioma. Meanwhile, chronic immune-mediated inflammatory disorders also contributed to carcinogenesis [43]. From this perspective, the fact that COPD is associated with an increased incidence of lung cancer may attribute to the chronic inflammation starting as lung barrier dysfunction.

## 3. EMT and EndMT in COPD and Lung Cancer

EMT is a crucial process during embryogenesis (type-1 EMT), which could also be induced as a result of continuous stimulus and inflammation [44]. Tissue fibrosis (EMT-type-2) and hypervascularity (EMT-type-3) are relevant to malignancy which is identified in one-third of the patients with metastatic lung cancer and in approximately 23% of non­small-cell lung cancer cell (NSCLC) lines [45, 46]. Both of the two latter processes may be correlated with pathology of cancer issue from COPD.

Classical EMT is usually described as the transition of epithelial cells to cells with a mesenchymal phenotype by the identification of prototypical markers such as E-cadherin and vimentin [46]. In the airways, EMT has been implicated in hypertrophy, metaplasia, gene mutation, and modification of lung epithelial cells which is considered as a critical mechanism in the transformation or pathogenesis of COPD [2]. In a previous study, there was an obvious increase in the number of blood vessels related to the reticular basement membrane. These vessels usually penetrated up into the epithelium in bronchial biopsies of cigarette-associated COPD patients [47]. The level of staining for vascular endothelial growth factor (VEGF) in newly generated vessels increased in both smoker groups and the COPD ex-smoker group, which indicated that EMT was an active process in cigarette-associated COPD and reticular basement membrane (Rbm) hypervascularity was related to active EMT in the pulmonary lesions [48]. In addition, a follow-up study revealed that vessels in the Rbm were more permeable than those normal vessels [49]. These evidences suggested that EMT in small airway was closely related to scaring and fibrosis and the consequent obstruction [50], while Rbm-related hypervascularity may contribute to the pathogenesis of the lung cancer predominantly observed in the airway compartment [51].

Type II alveolar cells are responsible for the synthesis and secretion of surfactant to decrease surface tension. Previously, type II alveolar cells were not involved in EMT or adopt mesenchymal features in wound healing process* in vivo* [52]. However, it is confirmed that CS could induce EMT in the alveolar type II cell line A549 [53], the bronchial epithelial cell line BEAS2B [54, 55], 16HBE [56], and primary human bronchial epithelial cell of smokers with COPD in vitro [2]. What is more, upregulation of phenotypic markers of EMT such as matrix metallopeptidase-9 (MMP-9), vimentin, and nicotinamide adenine dinucleotide phosphate oxidase-4 (NOX4) was found in bronchial epithelial cells of both large and small bronchi from smokers and COPD patients [57]. The level of transforming growth factor *β*-1(TGF*β*-1) staining in vessels was especially noticeable in the Rbm of smokers and in patients with COPD. Additionally, there is evidence of the activation of Sma-and-Mad-related protein (SMAD 2/3) pathway in current-smoking COPD subjects [58–60]. These studies implied that smoking induced EMT process in healthy airway epithelium* in vivo* prior to clinical manifestations of lung cancer.

Recent studies demonstrated that E-cadherin and *β*-catenin were reduced in epithelial cells those derived from COPD patients exposed to repetitive CS [15]. This indicated that the regulation of EMT revolved around the transcriptional suppression of E-cadherin and *β*-catenin, as well as the archetypal adhesion molecules. E-cadherin encoded by CDH1 is a key transmembrane protein in the adhesion junction complex. It is inserted in the membrane to mediate cell adhesion. The extracellular domain of E-cadherin was connected to the adjacent cells and the intracellular domain attached to the cellular actin cytoskeleton. The degeneration of cell-cell adhesion affected its interaction with the actin cytoskeleton and led to the initiation of intracellular signaling cascades subsequently and barrier dysfunction of monolayer. The mRNA expression of typical mesenchymal markers *α*-smooth muscle actin (*α*-SMA), vimentin, and collagen type 1 was significantly upregulated and epithelial marker zonula occludens-1 (ZO-1), while E-cadherin was downregulated in smokers and patients with COPD [61]. These results confirmed that EMT acted as an important part in the development of COPD. It has been widely acknowledged that a partial-EMT status assumed the adaptability of cells to proceed or reverse the EMT process [62]. The mechanism of CS exposure induced EMT is still under elucidated. One of the most likely interpretations is that cigarette smoke induces EMT in bronchial epithelial cells via release and autocrine action of TGF-*β* and by enhanced oxidative stress in the meantime. To date, a partial understanding has gained about the complex mechanisms that how TGF-*β* signaling is constitutive in EMT [63], the most well-known mechanism is that Smad-mediated transcription regulation is activated in TGF-*β* induced EMT [64, 65], and attenuating TGF-*β* signaling pathways has been the main strategy to withstand cancer associate fibrosis [66].

EMT-activating transcription factors (EMT-TFs) play critical roles in all stages of cancer progression including initiation, in situ tumor growth, invasion, metastasis, and colonization, as well as chemotherapy and radiotherapy resistance [44]. Nowadays, the widely studied TFs include Snail, Twist, and Zinc finger E-box-binding homeobox (ZEB) families which regulate transcription of membrane adhesion [67]. Other signaling pathways such as Wnt-*β*-catenin and phosphoinositide 3 kinase-protein kinase B (PI3K-AKT) could increase Snail 1 activity by preventing its phosphorylation by glycogen synthase kinase-3*β* (GSK-3*β*), which then enhanced the EMT [68]. It is already confirmed that Twist expression reduced epithelial gene expression and activated mesenchymal gene expression while intracellular Twist activity was amplified by its phosphorylation by mitogen-activated protein kinase (MAPK) [69]. Signal transducer and activator of transcription 3 (STAT3), one of the important downstream mediator of TGF-*β*1 signaling, is reported to be associated with the enhancement of lung biopsies from idiopathic pulmonary fibrosis patients and in mice with fibrotic lungs. STAT3 could also provoke EMT in collaboration with active K-Ras by increasing Snail expression in cancer cells [70]. What is more, in epithelial origin solid cancers such as lung, breast, and ovarian carcinomas, the range of intrinsic EMT gradients was more unbridled [71]. In lung cancer cancers, epidermal growth factor receptor (EGFR) mutation or anaplastic lymphoma kinase (ALK) fusion transcript status should be considered in conjunction with their intrinsic EMT process. The cross-talk between abnormal constitutive activation of STAT3 and EGFR was discussed recently. Over-reacted STAT3 contributed to the pathology of chronic inflammatory diseases and cancer. It was not inhibited by the continuous presence of the main negative regulator Suppressor of cytokine signaling 3 (SOCS3) since STAT3 was rephosphorylated by activated EGFR [72]. These results highlighted that STAT3 and EGFR might work synergistically in the transitory stage from COPD to lung cancer.

Another potential mechanism for epithelial cancers in COPD is endothelial mesenchymal transition (EndMT). In this process, endothelial cells lose their apical-basal polarity and adhesion properties to form highly aggressive, migratory, extended mesenchymal cells and conduce to different pathological processes [73]. EndMT has been proved as a vital process in heart development, cardiac fibrosis and bleomycin-induced pulmonary fibrosis [74]. EndMT could also result in tumorigenesis [75, 76]. It was reported that during EndMT procedure myofibroblasts and cancer-associated fibroblasts (CAFs) were produced. Meanwhile, the myofibroblasts and CAFs were generated by extracellular matrix molecules, while the CAFs could lead to production of extracellular matrix and paracrine factors resulting in generation of tumor growth and cancer progression. These findings could interpret the potential pathology as the tumor tissue was closely related to hyperangiogenesis [45, 75, 77, 78]. EndMT can also activate the formation of procancer stroma, which was quite similar to that seen with EMT-type-3. Videlicet, it has the potential to initiate cancer and contribute to proliferation of cancer cells [45]. Although current evidence suggested that EndMT and EMT may share some common signaling pathways, there are still some distinct differences. In particular, the expression of TJs, cytoskeletal proteins, and specific surface markers in endothelial cells was clearly different from these of the epithelial cells [78] (Table 1). TGF-*β*, Notch, and bone morphogenic protein (BMP) pathways were considered to play important roles in EndMT. Little is known about whether these pathways provide the initiating signal under physiologic conditions* in vivo* [76, 79–81], and further studies are required to investigate the roles of transcriptional networks in mediating EndMT downstream of these pathways. It was noteworthy that an increased expression of the Snail family of transcriptional repressors [82, 83] was observed during EndMT, where they were believed to make a crucial difference in disrupting cell-cell junctions by downregulating VE-cadherin. Meanwhile, increasing interests have been paid on the role of STAT3 in EndMT. Although it was reported that the activation of JAK/STAT3 pathway in endothelium cell line was associated with barrier dysfunction and the activation of STAT3 integrated common profibrotic pathways, decreased STAT3 could induce TGF-*β*1-mediated endothelial fibrosis [84, 85]. In future, further discussions are needed to investigate the mechanism of these controversial effects.

Since endothelium located in immediate could contact with the bloodstream, therapies focusing on inhibiting EndMT make it an attractive strategy for drug delivery, which may be widely applicable to various diseases. Due to the similarity between EMT and EndMT, we have reasons to take the two issues as a whole. Taken together, it is reasonable to assume that preventing EMT and EndMT in COPD may delay disease progression and allow patients to maintain prolonged status of competent lung function. Also, it may reduce tumorigenesis associated with CSE induced COPD.

## 4. Tissue Repair and Remodeling

CS has systemic effects in addition to altering lung function. COPD is particularly associated with systemic comorbidities such as coronary artery disease and other cardiovascular disorders [86, 87]. Recent researches highlighted that CS was also associated with intestinal dysfunction [88] and type II diabetes mellitus [89]. Circulating endothelial microparticles (eMPs) are thought to shed into the blood stream from activated, apoptotic, or necrotic endothelial cells. Blood eMPs are significantly elevated in healthy smokers and patients with COPD [90–92], which indicated that toxic constituents generated by both mainstream and secondhand smoke exposure were absorbed from the airways into the blood causes systemic toxicity subsequently [16], among which pulmonary endothelium played a vital role in these processes. The repair of vascular endothelium seems important in response to CS stimuli. As mentioned above, pulmonary endothelial denudation has been reported in smokers with COPD [20] and endothelial progenitor cell dysfunction has been recognized in patients with progression of COPD, suggesting that CS-induced inadequate repair of the endothelium may also contribute to COPD mortality [93]. In fact, the biomarkers of altered endothelial function have also been detected in some studies. In a multicenter cohort study, pneumonectomy specimens were obtained from patients with solitary nodules, who either had COPD, were smokers with normal lung function, or were nonsmokers with normal lung function, in order to identify the markers from the endothelial cells. In these patients, expression of angiopoietin-2 (ANGPT-2) was significantly associated with increased pulmonary artery vessel wall remodeling [94]. In another cohort, they found that plasma levels of ANGPT-2 were higher in current smokers than in former smokers, and COPD patients overall had higher levels of ANGPT-2 than nonsmokers. These results were demonstrated in a dose-response manner with pack-years [95]. Since ANGPT-2 was secreted by vascular endothelial cells in Weibel-Palade bodies, vascular remodeling had also been widely reported in mild to severe cases of COPD [77, 96, 97]. These findings sustained the assumption that CS altered endothelial cell function in cigarette-associated COPD patients.

To our knowledge, tumor tissue is heavily associated with increased angiogenesis which forms an abnormal vascular network characterized by disorganized, immature, and permeable blood vessels [98–100]. Targeting tumor angiogenesis and vascular normalization is also a considerable therapeutic strategy in lung cancer [101], breast cancer [102], colorectal cancer [103], and gastric cancer [104]. The mechanism of tumor vascular formation is rather complicated, among which VEGF, angiopoietins, platelet-derived growth factor (PDGF-B), and TGF-*β* families [105] played the leading roles. Angiopoietins play pivotal roles though a tyrosine kinase receptor (Tie2) which typically expressed on vascular endothelial cell and specific macrophage subsets [106]. The auto-phosphorylation of Tie2 leads to endothelial cell migration [107, 108], tube formation, sprouting, and survival [109, 110] via intracellular signaling pathways. It is noteworthy that, in the context of tumor angiogenesis, ANGPT-2 inhibition shows potential therapeutic benefits in certain circumstances [111–113]. Taken together, we believe that ANGPT-2 is a key signal in the process of COPD related malignant transformation, and this hypothesis still needs further research with* in vitro* model.

## 5. Conclusion and Perspectives

**Figure 2:**
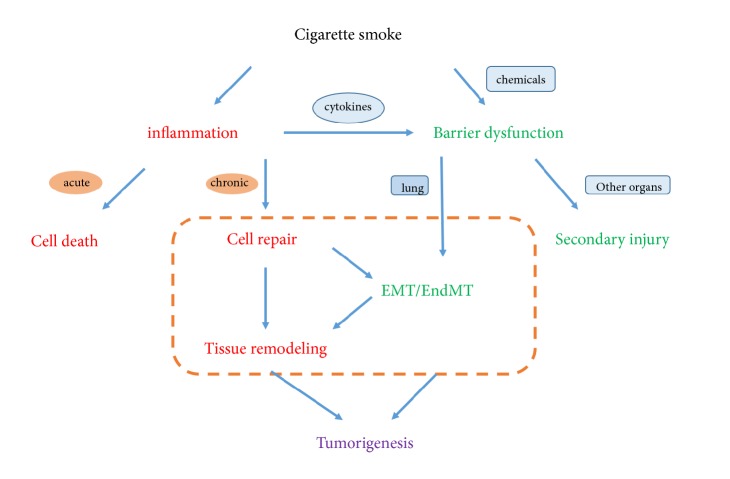
The complicated reaction caused by prolonged cigarette exposure. The part in the orange box is a possible mechanism between CS-induced COPD and lung cancer.

In summary, we highlight the complexity of pathways involved in CS associated COPD and lung cancer. Many persuasive mechanisms have been put forward to explain the causes or reasons for it. It has been well acknowledged that CS associated COPD should be considered as an important risk factor for lung cancer. In this article, we proposed a supposed mechanism of lung carcinogenesis from cigarette-associated COPD (Figure 2). The CS exposure caused lung barrier dysfunction and inflammation. Endothelium injury is a vital mediator in this process. The protracted inflammation gave rise to EMT and EndMT that end up with aberrant tissue repair. It might help interpret the link between COPD and lung cancer in smokers and may lead to the development of diverse strategies for the general management of COPD and lung cancer. Besides,* in vitro *model of cocultured epithelial cells and endothelial cells that able to mimic the microenvironment of alveolus may provide evidence for the interaction between epithelial and endothelial cells.

## Figures and Tables

**Table 1:** Comparison of epithelial endothelial and mesenchymal cells.

	Epithelial cell	Endothelial cell	Mesenchymal cell
junctions	AJs, TJs, Desmosomes w/E-cadherin	AJs, limited TJs, W/VE-cadherin	-
Barrier function	++	+	-
Intermediate filament	Cytokeratin	Vimentin	Vimentin
Markers	Claudins Occludins Cytokeratin Mucin-1 E-cadherin	CD31 VE-cadherin VEGFR Tie	*α*-SMA vimentin FSP1 Vitronectin
